# Exploratory Investigation of Motor and Psychophysiological Outcomes Following VR-Based Motor Training with Augmented Sensory Feedback for a Pilot Cohort with Spinal Cord Injury

**DOI:** 10.3390/bioengineering12111266

**Published:** 2025-11-18

**Authors:** Raviraj Nataraj, Mingxiao Liu, Yu Shi, Sophie Dewil, Noam Y. Harel

**Affiliations:** 1 Department of Biomedical Engineering, Stevens Institute of Technology, Hoboken, NJ 07030, USA; mliu26@stevens.edu (M.L.); yshi30@stevens.edu (Y.S.); sdewil@stevens.edu (S.D.); 2 Movement Control Rehabilitation (MOCORE) Laboratory, Altorfer Complex, Stevens Institute of Technology, Hoboken, NJ 07030, USA; 3 Spinal Cord Damage Research Center, James J. Peters VA Medical Center, Bronx, NY 10468, USA; noam.harel@mountsinai.org; 4 Departments of Neurology and Rehabilitation & Human Performance, Icahn School of Medicine at Mount Sinai, New York, NY 10029, USA

**Keywords:** virtual reality, neuromotor rehabilitation, spinal cord injury

## Abstract

Spinal cord injury (SCI) impairs motor function and requires rigorous rehabilitative therapy, motivating the development of approaches that are engaging and customizable. Virtual reality (VR) motor training with augmented sensory feedback (ASF) offers a promising pathway to enhance functional outcomes, yet it remains unclear how ASF modalities affect performance and underlying psychophysiological states in persons with SCI. Five participants with chronic incomplete cervical-level SCI controlled a virtual robotic arm with semi-isometric upper-body contractions while undergoing ASF training with either visual feedback (VF) or combined visual plus haptic feedback (VHF). Motor performance (pathlength, completion time), psychophysiological measures (EEG, EMG, EDA, HR), and perceptual ratings (agency, motivation, utility) were assessed before and after ASF training. VF significantly reduced pathlength (−12.5%, *p* = 0.0011) and lowered EMG amplitude (−32.5%, *p* = 0.0063), suggesting the potential for improved motor performance and neuromuscular efficiency. VHF did not significantly improve performance, but trended toward higher cortical engagement. EEG analyses showed VF significantly decreased alpha and beta activity after training, whereas VHF trended toward mild increases. Regression revealed improved performance was significantly (*p* < 0.05) associated with changes in alpha power, EMG, EDA, and self-reported motivation. ASF type may differentially shape performance and psychophysiological responses in SCI participants. These preliminary findings suggest VR-based ASF as a potent multidimensional tool for personalizing rehabilitation.

## 1. Introduction

Neurological trauma, such as spinal cord injury (SCI), can severely impair motor function, particularly in the upper extremities, leading to loss of independence in activities of daily living and compromised quality of life [1,2]. Affected individuals typically prioritize regaining functional use of the upper extremity among their rehabilitation and recovery goals [3]. Traditional physical therapy remains the clinical standard for motor rehabilitation, but its intensive and repetitive nature can be monotonous, fatiguing, and insufficiently engaging, especially for those with SCI. Such rehabilitation approaches can limit the gains in function over time [2,4]. Advanced technological platforms such as virtual reality (VR) are emerging as promising alternatives or complements to standard therapy by offering immersive, interactive, and gamified training environments that better sustain user motivation [5,6,7]. While VR is an effective delivery vehicle for rehabilitation, its full potential lies in its leading feature of customizability, specifically, in how sensory feedback is structured to guide motor training for individual users. Thus, it is paramount to verify how those with neurological traumas like SCI respond to systematic changes in sensory-based guidance provided in VR to strategically maximize effectiveness.

A high-potential pathway for customizing VR motor training is augmented sensory feedback (ASF), where sensory-driven cues (e.g., visual, haptic) are delivered in real-time to encode performance feedback to participants and support learning of goal-directed movements [8,9,10,11,12,13]. ASF has been shown to facilitate learning by providing real-time guidance that promotes motor error correction through spatial orientation [8,14,15,16,17]. Visual feedback is especially effective for visually guided tasks [8], while haptic feedback (e.g., vibration) has therapeutic advantages for enhancing proprioception, especially among individuals with sensory deficits [13]. Furthermore, multimodal ASF, whereby cues activate multiple sensory modalities concurrently, can reinforce guidance signals and accelerate motor learning by amplifying neural response [18] and crossing neural activation thresholds more readily [19]. Individuals with SCI often experience compromised somatosensory function while retaining relatively intact visual processing [20]. Furthermore, SCI affects the interplay of visual and somatosensory representations in the body [21]. Thus, it is unclear whether multimodal ASF can still enhance motor performance after SCI through the neural mechanisms typically associated with multimodal feedback, including visual and haptic feedback.

Regardless of the presence of SCI, the potential benefits of ASF to realize gains in function, like physical therapy itself, can depend critically on its dosage and delivery strategies. For example, providing excessive feedback can produce cognitive overload by overwhelming attentional and working memory resources [22,23]. Moreover, while rehabilitative methods must facilitate greater physical effort, i.e., “no pain, no gain” [24], it is crucial to mitigate premature fatigue, both physical and mental, that could precipitate injury or limit the capacity to regain better function [25]. Optimal rehabilitative training is achieved when individuals are sufficiently engaged but not exhausted by controllable training elements such as difficulty [26,27], and, in this case, feedback guidance [23]. To optimize VR motor therapy with ASF for individuals with SCI, it is important to examine their responses to different types of ASF, which can be innovatively customized. Responses of interest not only include task performance, but also psychophysiological measures that reflect bodily and cognitive states critical for motor training outcomes.

A variety of psychophysiological measures indicating factors such as neural engagement, emotional arousal, bodily effort, and subjective perceptions correlate to better performance [28,29] and can be leveraged for improving rehabilitative training. Skin-surface measures of brain, heart, skin, and muscle function can reliably monitor physiological responses to concurrent rehabilitative training in VR. Electroencephalography (EEG) measures within virtual environments can indicate user experiences related to mental workload and attention [30] or emotional arousal [31]. Heart rate (HR) and its variability are highly suggestive of sympathetic nervous system activation and serve as proxies for cognitive and emotional load [32]. Electrodermal activity (EDA) to assess emotional arousal has been demonstrated with specific features of computerized interfaces, e.g., 2D vs. 3D [33], and for evaluating ‘presence’ in VR [34]. Electromyography (EMG) is a direct correlate to the physical (muscular) effort related to a motor task [35].

Regarding perceptual measures, a sense of agency or perception of voluntary control is particularly intrinsic to human movement actions [36]. A sense of agency has been shown to be shifted with those with pathological cognitive and motor conditions [37,38], and it has been examined extensively with computerized interfaces like VR, where experiences of agentic control and embodiment can be systematically altered [39,40]. Our own work has demonstrated positive correlations with measures of agency and motor performance in computerized interfaces [41,42,43]. Agency has also been shown to improve over the course of motor rehabilitation [44]. Feelings related to motivation [45] and utility [46] are further hallmark perceptual measures to consider when evaluating the potential clinical effectiveness and acceptance of rehabilitation training approaches.

Adapting rehabilitative training based on monitored psychophysiological states is not performed as standard. More typically, adaptive training schemes adjust training difficulty based on rate of performance progress for each person [26,27]. Still, programmable computerized interfaces offer a unique pathway to more precisely personalize training parameters. Moreover, such personalization can be performed readily according to measures from skin-surface recordings and surveys, which represent a wide array of psychophysiological responses. A vital step to realizing such approaches for personalized training is understanding how individuals, especially those with neuro-injuries, respond at fundamental and psychophysiological levels to customizable training elements, such as ASF guidance cues in VR.

To address this gap, the current study preliminarily examines how motor performance and psychophysiological states vary for persons with SCI under two modes of ASF during a VR-based upper-limb motor training task. A custom platform was employed in which SCI participants commanded a virtual robotic arm to contact various targets in a VR environment via semi-isometric upper-body muscle contractions. During training trials, participants received either visual ASF (unimodal) or visual plus haptic (multimodal) ASF. A control condition with no ASF was not pursued to ensure SCI participants were not fatigued within a single session. Further justifying this approach in our prior works having demonstrated how ASF training consistently produces significant improvements in motor performance compared to a no-ASF control condition [47,48]. Confirming that post-training effects were similarly independent of task repetition was beyond the scope of the present work, which supported single, time-restricted experimental sessions for each SCI participant. As such, this pilot study was designed to examine relative psychophysiological and motor responses between different ASF modalities (visual versus visual + haptic) in individuals with SCI, rather than re-evaluate the baseline efficacy of ASF training itself.

In this exploratory study, data were collected from five participants with chronic incomplete cervical-level SCI, and post-training effects were evaluated using a multimodal framework of measures that included the following:

- Motor performance metrics: Reduced end-effector pathlength and reduced trial completion time indicate better performance [49].
- Electroencephalography (EEG): Changes in brain activity (frequency band powers) as indicators of cognitive and visuospatial loading during motor tasks [50].
- Electromyography (EMG): Changes in amplitude across all muscle activity recordings used as a command signal to further indicate broad physical (neuromuscular) effort needed to perform the task [51].
- Electrodermal activity (EDA): Intra-trial shifts as proxies for emotional arousal and engagement [52].
- Heart rate (HR): As a measure of cardiovascular exertion during physical training [53].
- Perceptual surveys: Self-reported measures of agency [54], motivation [55], and utility [56] suggest higher-order user perceptions of the various rehabilitative training modes.

This study examines how motor performance in individuals with SCI relates to concurrent changes in EEG, EDA, HR, and perception, as well as how these interactions vary with the type of ASF (unimodal versus multimodal) provided. By moving beyond motor metrics alone, this study also highlights how training feedback can be personalized to optimize performance while managing cognitive and physical demands.

## 2. Materials and Methods

### 2.1. Experimental Overview (Design, Setup)

**Figure 1:**
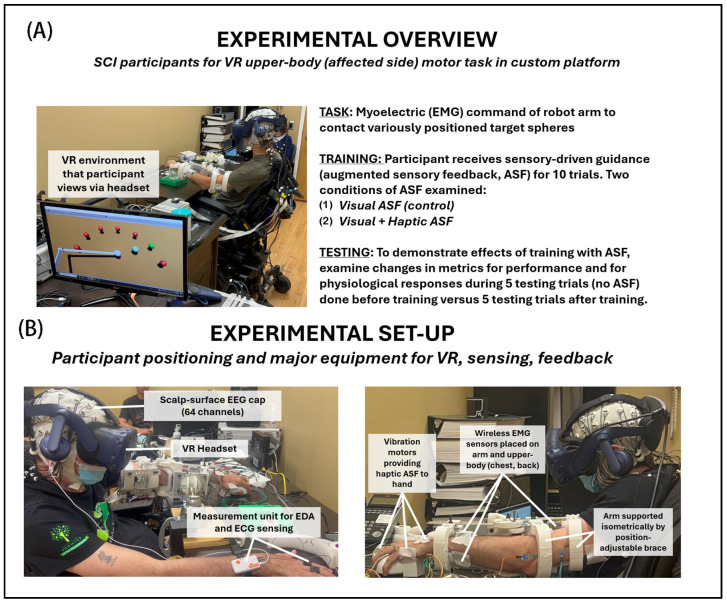
Experiment details: (**A**) overview (procedures, conditions) and (**B**) setup (equipment). Participants with incomplete cervical-level spinal cord injury had the affected arm in a position-adjustable support brace that provided resistance to movements. The participant used muscular exertions to semi-isometrically command (direction and speed) the end-effector of the robot arm to contact the targets within the VR environment.

The experimental outline to investigate upper-extremity motor performance under various ASF conditions is illustrated in Figure 1 (Panel A—protocol points, Panel B—apparatus). Participants with SCI placed their dominant-side arm into a custom position-adjustable support brace with surface-mounted physiological sensors for recording EMG, EEG, EDA, and ECG. Myoelectric activity from participants served as input to a pattern classifier that was used to command the two-dimensional (front–back and left–right plane) position speed and direction of the robot end-effector in the VR space to contact highlighted targets. Participants were informed that performance was positively assessed according to the minimal pathlength trajectories taken in contacting the targets (primary metric) and the minimal time taken to complete each trial (secondary metric). The effects of various ASF training conditions were assessed according to the mean change in performance and physiological metrics from a block of testing trials (5 total) performed before (pre) training to another block of testing trials performed after (post) training. Each block of training trials (10 total) presents a form of ASF intended to engage and provide guidance during training.

In general, ASF was provided to reinforce the best direction (shortest distance) to reach a given target. With the visual ASF, the cue was provided as a guide sphere, consistently placed between the user-controlled end-effector and the active target, and also progressively changed color with proximity to the active target. The same information was encoded with haptic ASF cues in the form of a vibration at the hand to denote the optimal direction (toward target), with the magnitude of vibration also changing (reducing) progressively with distance to the target. Each block of ten training trials included one of two feedback modes, either visual feedback (VF) only (unimodal ASF) or combined visual and haptic feedback (VHF) provided concurrently (multimodal ASF). The blocks of five pre- and five post-training trials were conducted without ASF. This protocol design allowed for the quantification of potential motor learning gains and neurophysiological engagement induced by the different ASF modalities used for training.

### 2.2. Participants

Five participants with chronic (greater than one year) incomplete cervical-level (C4–C6) spinal cord injury participated in this study. A prior pilot analysis (the study completed by [9]) with able-bodied persons using this platform suggested that *n* = 5 is sufficient to show significant difference (α = 0.05, 90% power) for MANOVA across three condition groups and two performance metrics (completion time, motion pathlength). This justified the sample size as proposed for the funded study (VA SPiRE Award# RX003582). The participant group included four males (aged 21, 25, 58, 61) and one female (aged 51). All of these participants had some experience with physical therapy, but no regular exposure to VR-based methods. Participants were clinically evaluated (International Standards for Neurological Classification of Spinal Cord Injury, ISNCSCI) [57]. Inclusion criteria for subjects included the following: (1) aged from 18 to 65 years, (2) post-SCI for more than 1 year, or (3) SCI between C1-T1, (4) ISNCSCI hand weakness scores ranging from 2 to 4 out of 5 manual muscle test in intrinsic hand muscles of thumb or 5th digit. Participants were excluded if they self-report any of the following: (1) any prior experience with a similar VR brace system or myoelectric control, (2) prior diagnosis of impaired cognitive abilities, or (3) prior history of major (requiring surgery or physical therapy) musculoskeletal injury to the upper extremities. Participants were not screened or excluded based on socioeconomic or educational background, in accordance with VA research principles of equitable access and inclusion. All participants were provided with, and signed, informed consent forms. This protocol (#IRBNet 1591774) was approved by the Institutional Review Board at the James J. Peters VA Medical Center. The brace was configured for each participant’s arm on the affected side, with preference to the dominant-side arm. The brace was configured for the left side for three participants and the right side for the remaining two.

### 2.3. Skin-Surface Sensors for Physiological Recordings

Real-time electromyography (EMG) data were recorded using 14 wireless sensors (Trigno, Delsys Inc., Natick, MA, USA) from upper-limb and trunk muscles: brachioradialis, extensor digitorum, biceps brachii, triceps brachii, upper/middle/lower trapezius, infraspinatus, serratus anterior, latissimus dorsi, pectoralis major, and anterior/lateral/posterior deltoids. EMG was sampled at 1728 Hz. Electroencephalography (EEG) data was recorded using a 64-channel actiCHamp Plus amplifier (Brain Products GmbH, Gilching, Germany) at a sampling rate of 5 kHZ (re-sample for analysis at 256 Hz). EEG power spectral density was computed offline in delta (0.5–4 Hz), theta (4–7 Hz), alpha (8–12 Hz), and beta (13–30 Hz) bands. Electrodermal activity (EDA) and electrocardiogram (ECG) data were recorded using a Shimmer3 GSR+ system (Shimmer Inc., San Francisco, CA, USA), sampled at 51 Hz. EDA/ECG electrode leads were placed on the ear and index and middle fingers of the hand and ear on the side not involved with the brace platform. All physiological signals were synchronized offline, based on the recorded low–high signals generated to mark the beginning and ends of the individual trials for each system.

### 2.4. Upper-Extremity Brace Platform

A computerized rehabilitative interface with VR compatibility for training coordinated muscle contractions of the upper extremity, originally developed for Sanford et al. [9], was the experimental centerpiece for this study. This training platform primarily includes a custom designed 3D-printed arm brace with three main components: an upper arm/forearm mount, an adjustable rod, and a tabletop base mount. While supported in the brace, participants could generate directional forces with the upper extremity, from which EMG was recorded for myoelectric control of a virtual avatar. The brace allowed for gravity-supported positioning of the dominant-side arm to assume an arm posture within the following joint angle ranges: shoulder abduction/adduction (45–75°), shoulder internal rotation (0–45°), and elbow flexion (90–120°). When generating higher forces, there was some compliance (~2 cm maximum displacements) in the brace whereby muscular contractions were primarily isometric, but allowed for minimal displacement, functionally resembling quasi-isometric or low-range isotonic contractions. This compliance was beneficial in terms of participant comfort (more distribution of pressure across skin) and mitigating potential muscular strains, whereby changes in force direction combined with slight compliance presented more as a series of short-range isotonic contractions rather than as purely isometric [58]. However, the restriction within the brace was sufficient for participants to induce semi-isometric muscle contractions within their upper extremity for potential therapeutic effects, along with robust recording of EMG signals used for myoelectric control [59].

The brace was position-adjusted for each individual participant to be at an experimentally defined neutral position, requiring each participant to have their shoulder relaxed (not elevated) and the long axis of their forearm to point forward. From this neutral position, participants could generate forces across their supported, but restrained, preferred (but affected) side of their upper body (arm, chest, back) to intuitively and internally map the attempted movement of their dominant-side hand with the VR end-effector. The hand was supported by a 3D-printed structure, being lightly gripped (similar to a computer mouse) and readily sliding with small movements, helping to calibrate the hand and arm to be in a consistent position. Once the hand was in the support position, five small-vibration motors (ERM DC 11000 RPM, Vybronics Inc., Winter Park, FL, USA) were placed on the front (1 motor at the dorsal side of the middle finger), left (1, dorsal-side bottom of the thumb, assuming the right hand), right (1, the ulnar-side of the hand below the small digit, assuming right hand), and back (2 motors on the dorsal- and palmar-side of the wrist) of the participant’s reaching hand. These motors could be differentially activated to provide haptic feedback as directional and magnitude cues.

### 2.5. Virtual Reality (VR) Task Environment

A custom VR environment was developed in Unity for real-time myoelectric control of a virtual robot arm to contact various targets, running on a workstation computer with Intel Xeon CPU ES-1660 v4, 3.20 GHz, 32 GB RAM, Windows 10 Pro OS. The robot arm end-effector was spherical and connected to a two-segment linkage changing angles according to an inverse kinematics routine [60]. Each participant viewed the environment using an HTC Vive head-mounted display (HTC Corporation, Taoyuan City, Taiwan). The headset was placed over the EEG cap. Data from the VR environment (e.g., position data of the VR robot end-effector, guidance cue updates) were captured at 90 Hz. The VR environment received data regarding end-effector position from MATLAB 2024b (Mathworks, Inc., Natick, MA, USA) API that connected MATLAB toolboxes including those to train and run the participant-specific EMG pattern classifier. Based on arbitrary distance units for the VR space, the spherical end-effector and targets were specified as 1 unit in diameter, and the radial distance from the initial centered position of the end-effector within the semi-circular arrangement of training targets was 7 units. Thus, the range of possible inter-target distances for training trials was from ~3.6 (distance between adjacent targets) to 14 units (diameter of circular target arrangement). For testing trials, the inter-target distances were typically from 2 to 5 units, depending on the participant’s choice of sequence of targets to contact.

### 2.6. Individual Testing and Training Trials

**Figure 2:**
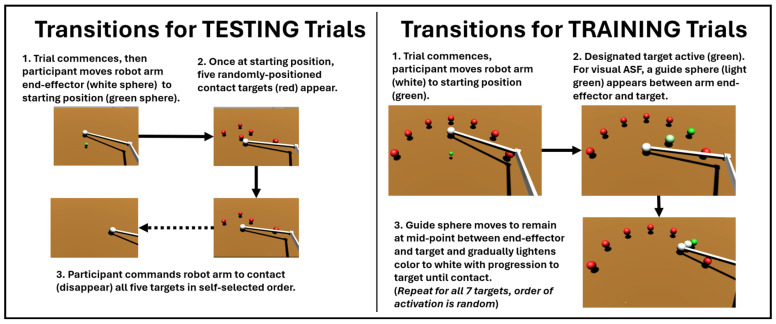
Details of SCI participants using myoelectric commands to transition robot end-effectors within the testing trials (LEFT), performed before and after training, versus the training trials (RIGHT), in which varying conditions for augmented sensory feedback (ASF) were applied. The effects of ASF are assessed based on the changes in variables during the testing trials from before (pre) to after (post) training.

The virtual reality (VR) interface is shown in Figure 2, highlighting the target-reaching task of a virtual robot arm moving along a 2D plane under myoelectric control for the testing and training trials. For the testing trials (Figure 2, left panel), after the trial was initiated (the end-effector was moved to the starting position), the participant commanded the robot arm end-effector to move and contact a random arrangement of five targets in their preferred order. Upon contact, the target disappeared. For the training trials (Figure 2, right panel), seven targets were initially positioned in a pre-determined semi-circular arrangement (equally spaced from 0 to 180 degrees). The end-effector starts at the central equidistant point and, from there, the participant must move towards the target designated as active (target color turns green, other targets remain red). The participant then continues contacting the designated targets one by one until all seven targets are contacted. The order of the targets being activated is unknown to the participant a priori. Participants were told that performance would primarily be evaluated based on minimizing the pathlengths taken by the end-effector in contacting the target, but also that they would further be evaluated by how quickly they contacted all of the targets in each trial. Thus, participants should have attempted to minimize pathlength as a priority, but still sought to finish contacting all of the targets in a timely manner.

### 2.7. Training of EMG Pattern Classifier Used for Myoelectric Control

Myoelectric commands were decoded using support vector machine (SVM) classifiers trained individually for each participant. Using the ‘fitrsvm’ command in the MATLAB Statistics and Machine Learning Toolbox, two separate support vector machine regression models (one model for each direction in 2D planar control of end-effector) were created for each participant. Each model would input processed EMG from all fourteen recorded locations. The raw EMG signal from each location was processed for amplitude using a root-mean square (RMS) function [61] and averaged over the preceding 100 ms prior to model input. The support vector machine outputs were two position shifts (left–right direction or front–back direction) from the current time to the next time instance. The regression feature allowed for continuum (non-integer) outputs, which facilitated speed control, whereby participants could move the end-effector faster with greater magnitude of muscular effort. The training data for the pattern classifier was collected as part of a preliminary set of trials within the VR environment. For each classier training trial, a participant needed to exert a certain percentage threshold of MVIC to move the end-effector (shown moving at constant speed) across a finish line in one of four directions (front, back, left, right). To cross the finish line, participants needed to exert above-threshold effort for three 2 s bursts (minimum of 1 s rest interval) in the given direction. Participants completed a total of eight trials for pattern classification training. One trial in each of the four directions was repeated at 20% and 40% MVIC thresholds. For classification purposes, the muscle activation data during above-threshold bursts were labeled as ‘1’ in the designated direction for that trial and ‘0’ for all other directions. A fixed total of 100 iterations were executed in training each pattern classifier, which took approximately 5–10 min using MATLAB release from 2023 on the same computer running the VR environment.

### 2.8. Experimental Protocol

Each participant completed a single experimental session lasting ~3 h, including de-briefing, setup, EMG pattern classification, and running trial blocks (pre-training, training, and post-training) for each of the two ASF training conditions. Setup time (~30 min) included positioning the brace and fitting (custom placement of padding) to each participant and placement of skin-surface sensors for physiological recordings. Another 30 min was devoted to running pattern classification. A few minutes were then spent on practice trials to familiarize the participant with using myoelectric control to command the avatar position. As part of these practice trials, each participant was provided the opportunity to adjust the gain on their interface such that they perceived movement of the avatar as being well controlled. The gain was a multiplier on the distance the end-effector would move for a given pattern of myoelectric inputs in each directional dimension. The participants were allowed to choose from the current default gain of 1 (i.e., no change following classifier training) or a gain that was 50% greater (1.5) or 50% lower (0.5) than the default gain. The remaining session (~2 h) was used to test effects when training with each of the two training ASF conditions. For each training condition, participants performed a sequence of three blocks of trials as follows: (1) pre-training block = five testing trials (i.e., 5 randomly arranged targets) with no ASF to establish baseline for all measures; (2) training block = 10 training trials in which a form of ASF was provided in contacting 7 targets in a semi-circular arrangement; and (3) post-training block: repeating the five testing trials and evaluate the effects of training by comparing shifts from baseline for all measures. Each testing and training trial took approximately 10 and 30 s, respectively, to complete. The two training conditions for which the three-block sequence was repeated were presented to each participant in random order (to mitigate potential order effects) with >10 min breaks between conditions to use for rest and washout. The two ASF conditions were as follows:

1. **Visual ASF (Unimodal)** = A guide sphere was presented as moving continuously at the variable midpoint between the moving end-effector and the fixed active target. The strategic intention for guidance was to provide participants a target that was effectively closer in which to visualize a shorter pathlength and project motor actions that were still directionally optimal in progressing towards the final target. The guide sphere provided further engagement through progressive color changes from green to white as the end-effector approached the final target. The distance-level range in color change was fully green at a distance of 14 units (maximum possible starting distance from a target) to fully white upon contact to a final active target. Color updates occurred at a VR update rate of 90 Hz.
2. **Visual plus Haptic ASF (Multimodal)** = In addition to the visual feedback, differential vibration cues were concurrently provided based on the frequency magnitude applied to the motors positioned on the right, left, front, and back portions of the hand. An attractive scheme was employed where participants felt a net vibration in the direction they should move the VR end-effector toward. Similarly to the progressive color with visual cues, the magnitude of vibration frequency progressively reduced to zero upon contact with the active target from its maximum (~183 Hz) when the end-effector was at a distance of 14 units from that target. The resolution of changes in haptic ASF change was less than that of visual ASF, as only five levels of vibration were effectively apparent across its full range.

### 2.9. Subjective (Perceptual) Assessments

Within each experimental session, and after each ASF training conditions, participants provided survey responses to rate their perceptions of that training mode on a scale of 0 to 100 regarding how much they agree (0 = fully/strongly disagree, 100 = fully/strongly agree) with the following statements:

- “I was in full control of the virtual robot arm during training” (sense of agency).
- “The mode of training was motivating” (motivation).
- “The mode of training was useful for improving performance” (utility).

### 2.10. Data Analysis

All statistical analyses were conducted in MATLAB upon key variables, taken typically as trial-level averages for each of the 5 participants for subsequent group evaluation across conditions. Prior analysis with neurotypicals suggested sufficient power (α = 0.05, 90% power). The present data suggested that *n* = 4 was sufficient for 80% power at α = 0.05. All performance and psychophysiological variables were evaluated according to the average percentage change from the block of testing trials conducted before a given training ASF condition to the testing trials conducted after training. This evaluation approach allowed for the assessment of the effects of each ASF training condition based on the changes in performance and psychophysiological responses when ASF was not present and participants were not reliant on any assisting guidance (i.e., testing trials). While evaluating such measures within training blocks have reporting value, the primary focus of this study is demonstrating the resultant immediate (short-term) effects of various modes of ASF training at the designated dosages after training.

The performance variables of interest were the end-effector 2D motion pathlength (primary metric) and trial completion time. The physiological measures were power amplitude of four frequency bands for EEG, Delta (1–4 Hz), Theta (4–7 Hz), Alpha (8–12 Hz), and Beta (13–30 Hz), at the primary motor cortex (M1); RMS amplitude of EMG averaged across all 14 muscle recordings; the intra-trial change in EDA measured as skin conductance; and heart rate as total beats inferred from ECG signal per trial divided by trial time. For perception measures, no mean-trial computations were taken, given that a single rating was provided by each participant for agency, motivation, and utility with each of the two ASF training conditions.

All statistical testing was performed using the Statistics Toolbox in MATLAB. The Kolmogorov–Smirnov test was applied to each variable of interest to suggest sufficient normality for parametric testing. Barlett’s test was applied to each variable of interest to suggest sufficient homogeneity of variance for parametric testing. For variables in which sufficient normality or homogeneity was not determinable, given the small sample size, then non-parametric tests were applied (Wilcoxon signed rank test for symmetric distribution). Otherwise, a one-way ANOVA (single factor: ASF training condition; effectively two-sample *t*-test given only two ASF conditions) was performed with each performance, physiological, and perceptual measure. One-sample *t*-tests were also applied to each measure for each condition to infer if a significant (non-zero) change from before to after training was incurred. To examine any broad potential dependency of the primary performance metric (motion pathlength), in this study, onto each of the psychophysiological variables, a linear regression was applied on the scatter plot of participant-level performance versus psychophysiological variable mean values. A significant dependence was evaluated based on the presence of a significantly non-zero slope of the fitted regression line.

## 3. Results

**Figure 3:**
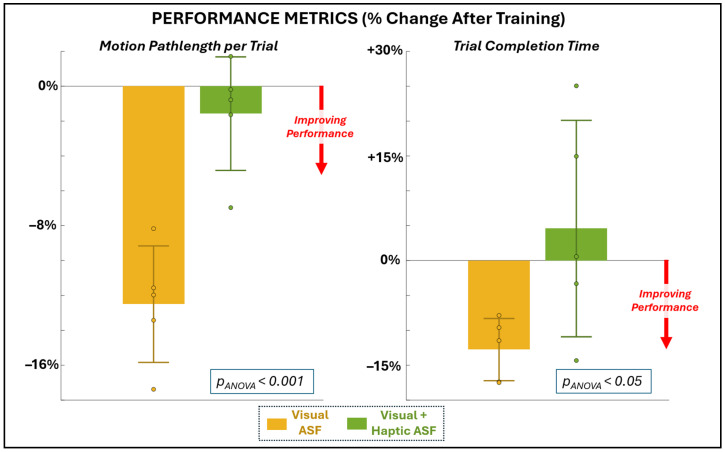
Changes in performance metrics of motion pathlength (primary metric, **left**) and completion time (secondary, **right**) as a percentage change from before to after training with each ASF condition.

The minimal data set for this study expressing mean values for each participant for all variables of interest is provided as Supplementary Materials. All of the variables of interest exhibited sufficient normality and homogeneity (*p* > 0.05, failing to reject the null hypothesis of normal distribution and homogenous variance, respectively), except for a select few variables that failed at least one of these tests. These variables included the performance measure of completion time (failed normality), the physiological measures of EEG delta and theta band power (both failed homogeneity), and the perceptual measure of utility (failed normality). These measures were additionally evaluated using non-parametric tests. Figure 3 and Table 1 summarize the changes in motor performance after training with visual (VF) and visual plus haptic (VHF) ASF. Only VF yielded significant (non-zero) performance improvements. Specifically, VF significantly reduced end-effector pathlength (−12.5% ± 3.3, *p* = 0.0011). Trial completion time data was evaluated using a non-parametric test, but still produced a significant non-zero difference (−12.8% ± 4.5, *p* = 0.031). In contrast, VHF produced non-significant changes from zero in both pathlength (−1.6% ± 3.3, *p* = 0.34) and completion time (+4.6% ± 15.5, *p* = 0.54). The Wilcoxon signed rank results confirm a significant difference between ASF conditions for pathlength, with VF outperforming VHF, but not for completion time.

**Figure 4:**
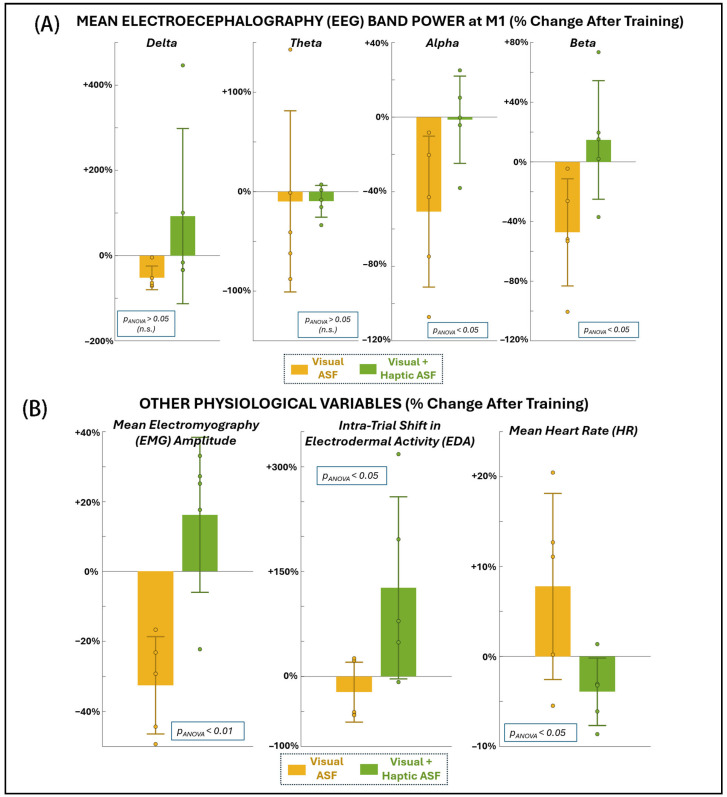
Changes in physiological variables as a percentage change from before to after training with each ASF condition: (**A**) EEG at the primary motor area (M1), (**B**) mean EMG across all muscle locations recorded at dominant-side chest, back, and arm (**left**), shift in EDA within the trial, i.e., from beginning to end of trial (**middle**), and mean HR overall (**right**).

Figure 4A and Table 1 present EEG power changes at the primary motor cortex across frequency bands. VF was associated with significant reductions in both alpha power (−50.7% ± 40.5, *p* = 0.049) and beta power (−47.3% ± 35.9, *p* = 0.042). Delta power also decreased significantly with VF (−52.3% ± 27.8, *p* = 0.031), even with non-parametric testing. In contrast, VHF showed no significant changes in any EEG band, although it trended toward increasing alpha and beta activity. Furthermore, the ANOVA results confirm that VF produced significantly greater reductions in alpha and beta activity in comparison to VHF. As shown in Figure 4B and Table 1, VF also produced a robust reduction in EMG amplitude across muscles (−32.5% ± 13.9, *p* = 0.0063). Since VR also improved motor performance, the concurrent reduction in EMG suggested improved neuromuscular efficiency. VHF, by contrast, showed a non-significant increase in EMG (+16.2% ± 22.2, *p* = 0.18), but the ANOVA confirmed the change in EMG with VHF was significantly different from that with VF. Neither condition produced significant within-condition changes in EDA or HR from pre- to post-training. However, the ANOVA revealed significant between-condition differences, with VF generally associated with reduced EDA and slightly elevated HR, and VHF showing the opposite pattern (increased EDA and reduced HR). These findings suggest that, while ASF training did not induce consistent autonomic shifts within either condition, the two ASF modalities elicited distinct autonomic response profiles.

**Figure 5:**
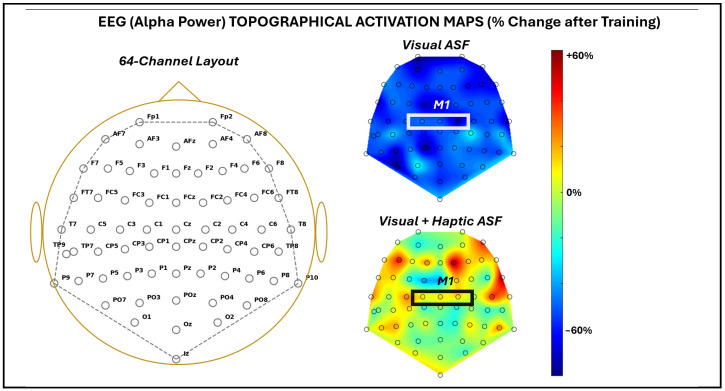
EEG activation map across 64 EEG channels recorded as average across all five SCI participants. For the given channel labels approximating the 10-10 convention (**left**), activation maps were provided as a percentage change from before to after training with each ASF condition (**right**). The approximate area of the primary motor cortex is highlighted by a white box. NOTE: Because the participants used either their left or right arm, EEG data from left-arm users were mirrored across the mid-sagittal axis before group averaging. This preserved hemispheric correspondence for the primary motor cortex and enabled consistent visualization across participants, while precluding interpretation of true lateralized asymmetries.

Figure 5 demonstrates the changes in the pan-cortical EEG alpha band power broadly observed across 64 channels after training via heat maps for each of the ASF training conditions. The mean change in alpha band power across all channels was −53 ± 13.3% for VF and 3.7 ± 22.2% for VHF. Across all of the channels, the reduction in alpha power with VF was significant (*p* = 1.8 × 10^−40^), and ANOVA confirmed a significant difference (*p* = 1.7 × 10^−35^) between the conditions.

**Figure 6:**
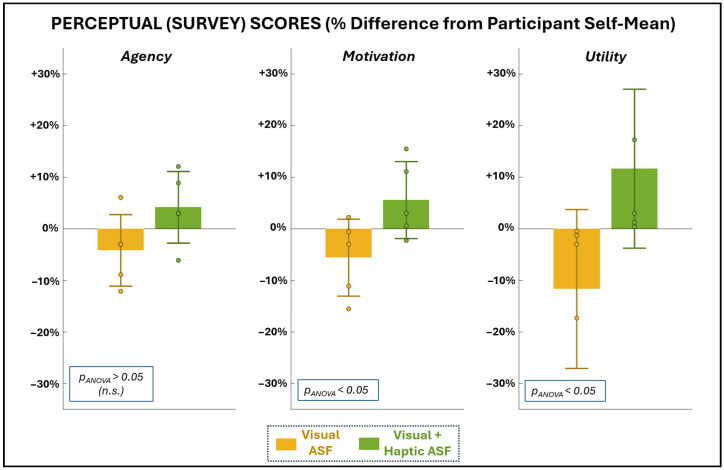
Average scores for the survey metrics used to assess SCI participants’ sense of agency, motivation, and utility with each training condition. Scores are presented as a percentage change from each participant’s own mean score (0–100 range) for given perceptual dimension to remove participant-specific magnitude effects and better isolate relative perceptual differences between ASF training conditions.

Figure 6 and Table 2 show the self-reported perceptions of agency, motivation, and utility following each ASF condition. Perceptual ratings are reported both as raw mean scores (0–100 scale) and as normalized percentage differences from each participant’s mean to account for individual response bias. No significant differences were observed between VF and VHF for agency (parametric evaluation) or utility (non-parametric evaluation). Trends in motivation favored VHF (both positive relative shifts), whereas VF trended negative. While these differences did not reach significance from each participant’s self-mean, the ANOVA results indicate a significant difference between the conditions for motivation.

**Figure 7:**
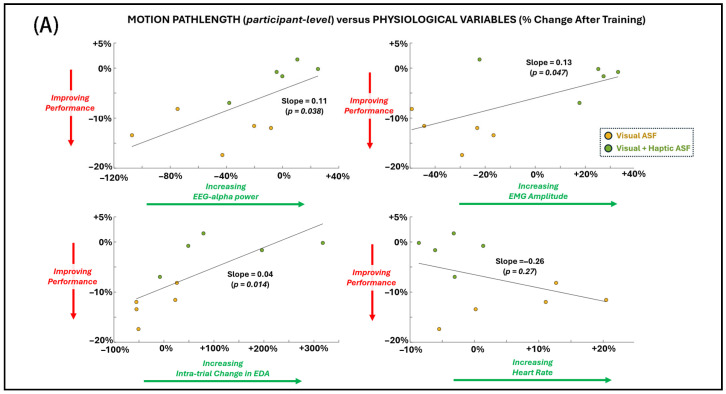

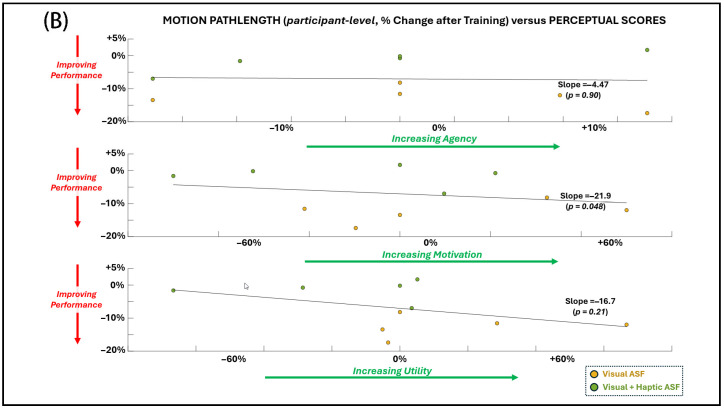
General dependence (across individual SCI participant values for both ASF conditions) of the primary performance metric (motion pathlength) on (**A**) physiological variables and (**B**) perceptual scores. Simple linear regression is applied across all points, and significance (alpha = 0.05) for non-zero slope is indicated by *p*-value.

Regression analyses (Figure 7A,B) demonstrated significant associations between changes in primary motor performance (shorter pathlength) and several physiological and perceptual measures across both ASF conditions. Improved performance was significantly (*p* < 0.05 for non-zero regression slope) related to decreased EEG alpha power, reduced EMG amplitude, reduced HR, and higher self-reported motivation. No significant relationship was found between the primary performance measure and EDA or perceptual responses for agency and utility.

## 4. Discussion

This exploratory study examines how unimodal (visual) and multimodal (visual plus haptic) augmented sensory feedback influenced motor performance, neurophysiological responses, and perceptual ratings in individuals with SCI during a VR-based motor training task. Similarly to Fabio et al. [62], who showed that technology-assisted cognitive rehabilitation can enhance engagement in patients, our study preliminarily suggests that immersive VR motor training with augmented sensory feedback may foster motor and psychophysiological improvements in individuals with spinal cord injury. The findings indicate that ASF type may differentially shape motor outcomes and underlying psychophysiological states for persons with neurological trauma. Still, these findings offer an insight into how ASF may be tailored in motor rehabilitative training after neurological traumas. Such training approaches could be readily employed using computerized interfaces with powerful options for customization and activating sensory modalities.

### 4.1. Motor Performance Effects of Unimodal Versus Multimodal ASF

Visual ASF produced significant improvements in both primary and secondary motor performance measures, i.e., reducing end-effector pathlength and trial completion time after training. These results align with prior work demonstrating that visual guidance strongly facilitates spatial orientation and error correction in motor tasks [8,14,63]. By contrast, the addition of haptic cues (VHF condition) did not yield reliable improvements. This suggests that, at least in the present SCI cohort, visual ASF alone provided more optimal guidance to support performance, at least for the primary metric of motion pathlength, whereas multimodal cues may have introduced additional cognitive load that offset potential benefits [22,23]. Individual variability may support multimodal ASF as more beneficial for some participants and justify personalized application. However, the findings of this study suggest adding concurrent haptic feedback appears to neutralize potential benefits of ASF for the SCI population in this rehabilitation task. In a prior study with this platform testing neurotypical individuals, training with VHF was similarly not as effective in improving post-training performance as with VF [47]; however, VHF training with neurotypicals still produced a significant (non-zero) improvement in that study.

Those with SCI typically increase reliance on visual feedback for motor tasking given compromised somatosensory function [64]. Adding haptic cues, which are less reliable after SCI, may introduce noise or extra cognitive demand that interferes with, rather than enhances, multisensory integration with ASF motor training [20,21]. Additionally, the haptic feedback implementation in this study used a fixed motor configuration and limited resolution in vibration levels, which may not have offered the necessary directional precision or perceptual salience of cues for individuals with impaired somatosensation. Thus, for those with neurological damage to benefit from multimodal ASF training, either somatosensory feedback may need to be customized to accommodate patient-specific deficits or ASF should target sensory modalities that are relatively uncompromised. Our previous work involving VR-based training for a grasp-and-place task suggested that those with traumatic brain injury needed additional cueing, i.e., visual plus audio, to improve post-training performance [10]. In this study, the cues were more simply provided as a singular cue as to when the participant achieved secure grasp, which contrasts with the more information-rich cues in this study whereby participants continuously received directional and amplitude indicators about error throughout the training task. Similarly with that platform, those with SCI also benefitted from more intensive sensory-based cueing, provided in immersive VR [65]. However, the ASF was provided through visual and audio channels, and the cues were simplified. Taken together, prior findings and those from this study suggest that, for individuals with SCI, ASF strategies may be more effective when they emphasize relatively intact sensory modalities—particularly vision and audition. This may be especially the case when engaging in training paradigms that entail continuous feedback that could otherwise overload cognitive resources and hinder motor learning.

### 4.2. Neurophysiological Modulation with ASF Training

The neurophysiological data further distinguished visual from multimodal ASF. Training with VF significantly reduced EMG activity, consistent with improved neuromuscular efficiency and reduced co-contraction [51,66]. Reducing co-contractions is a major objective in neuromotor rehabilitation following neurological injury, as it supports smoother and more coordinated movements [67]. VF also produced significant decreases in EEG alpha and beta power. Reductions in alpha and beta power with VF may indicate altered cortical activation patterns, reflecting either more efficient sensorimotor processing or reduced demand on sustained attentional resources. While decreased alpha power is commonly associated with increased cortical activation, emanating from higher cognitive and additional demands [30,50,68], in this context of improved performance and reduced EMG activity, decreases may suggest that using visual ASF cues only facilitated more efficient neural resource utilization rather than diminished engagement. In contrast, VHF training trended toward increased EEG alpha and beta power, consistent with imposing greater cognitive, but resulting in marginal changes in performance.

EDA and HR responses did not produce significant (non-zero) changes for either condition, although divergent trends were observed. VF tended toward lower EDA and slightly elevated HR, whereas VHF showed the opposite pattern (elevated EDA and reduced HR). Furthermore, these divergence phenomena for each metric produced a significant difference between conditions despite the limited sample size. Although neither condition independently produced significant post-training autonomic changes, the divergent HR and EDA patterns across ASF modalities indicate that visual and multimodal feedback may differentially engage autonomic regulation. More broadly, elevated EDA in the absence of higher muscular or cardiovascular effort can reflect sympathetic arousal linked to cognitive load or emotional engagement [33,52,69,70]. Such trends highlight that ASF type can uniquely modulate the balance of cognitive and autonomic responses with a rehabilitation training protocol.

### 4.3. Perceptual Ratings of Training Modes

Across both ASF conditions, participants reported generally high perceptual scores (mean scores > 75/100), indicating that both feedback modes were well received even when the normalized differences between conditions were modest. Perceptual ratings suggested differential subjective impressions of ASF modalities. Agency ratings relative to each participant’s mean did not differ significantly between VF and VHF. This observation suggests that participants generally perceived robust control of the VR arm, regardless of cue type. This is consistent with prior VR rehabilitation studies where the sense of agency is desirably stable [44]. By contrast, motivation and utility ratings trended higher for VHF compared to VF, consistent with the literature indicating higher acceptance of approaches that enhance user engagement and perceived task relevance [46,56]. However, in this preliminary study, this was only demonstrated as a significant difference for motivation. Regardless, in this study, this higher perceptual rating of engagement for VHF is in contrast to the VF case producing improved post-training performance, suggesting the need to carefully consider when user perceptions may contradict training approaches that produce better results.

### 4.4. Coupling Between Performance and Psychophysiology

Regression analyses revealed that improved motor performance was associated with concurrent reductions in EEG alpha power, EMG activity, and EDA. These findings support the notion that optimal motor performance emerges from an integrated state of efficient neuromuscular output, moderated cortical engagement, and balanced autonomic activation. There was also a significant (non-zero regression slope) relationship between improved performance and reduced motivation. However, this trend with motivation is not consistent with expected notions of improved performance with positive affective responses [62,71]. This finding suggests that, while the addition of haptic feedback was perceived as motivating, it paradoxically impaired performance, highlighting a dissociation between subjective appraisal of training and its actual functional benefit. Thus, while participant perceptions are critical for clinical acceptance of a rehabilitative approach, they may not always align with validating the efficacy of the approach in supporting functional recovery. Still, the small sample size of this study does may not definitively confirm this inverse performance–perception relationship for this study.

### 4.5. Implications for Adaptive VR Rehabilitation

The present results, which indicate the differential responses of SCI participants to different modes of ASF-based training, highlight opportunities for adaptive ASF-based rehabilitation tailored to individuals with SCI. Visual feedback may be prioritized when the goal is immediate performance improvement and efficient neuromuscular engagement. Conversely, multimodal feedback, despite less consistent performance benefits, may be valuable for enhancing engagement, attention, or perceived training relevance. Future adaptive systems could integrate psychophysiological monitoring (EEG, EMG, HR) to dynamically tune ASF parameters, balancing physical effort, cognitive demand, and motivation in real-time [32].

### 4.6. Limitations and Future Work

Although significant differences were shown for select metrics, this study involved a small sample of five participants with incomplete cervical SCI, limiting broader generalizability and increasing the risk of false-positive findings. While justified given our prior works and avoiding intra-session fatigue with SCI participants, this study did not carry out a no-ASF training condition, nor was it able to evaluate the effects of haptic-only ASF for this group. Future studies should incorporate a no-ASF control condition to confirm whether the observed changes stem from ASF-specific mechanisms rather than generalized task repetition or time-on-task effects for those with SCI. Currently, the results are exploratory, and the observed differences reflect within-participant responses to ASF modality rather than the verified efficacy of ASF itself for the population.

Also, the haptic ASF in this study was carried out with motors that are relatively lower resolution compared to the visual ASF cues readily provided within the VR environment. The haptic ASF used in this study employed fixed motor placements and limited resolution in vibration intensity, which may have restricted the fidelity of directional cueing. Future designs should calibrate haptic amplitude and spatial mapping to individual sensory thresholds to better accommodate post-injury somatosensory deficits and enhance multimodal integration. Furthermore, the evaluation of training effects was restricted to single-session exposure, preventing conclusions about longer-term retention or cumulative rehabilitation potency. Future work should employ longitudinal designs to examine whether ASF-specific gains translate into demonstrable functional recovery. Additionally, closed-loop VR systems that automatically adjust ASF intensity or modality based on physiological states of individual patients could provide more personalized rehabilitation trajectories [32,72,73]. Interpretations of EEG modulation in this study should also be regarded as preliminary, given the small sample size and lack of region-specific or connectivity-based analyses that could better delineate the neural mechanisms underlying ASF-related effects.

Consistent with smaller-scale investigations, such as Fabio et al. [62], who emphasized engagement and feasibility rather than therapeutic efficacy, the present study should likewise be interpreted as exploratory. The observed behavioral and psychophysiological changes may indicate responsiveness to ASF-based VR training, but cannot establish causal benefit without appropriate control conditions or larger cohorts. These early findings, however, help to delineate measurable outcome domains—motor, cortical, autonomic, and perceptual—that can be systematically evaluated in future controlled or longitudinal rehabilitation trials involving clinical populations.

## 5. Conclusions

Visual ASF significantly enhanced motor performance and reduced neuromuscular effort in SCI participants. Although multimodal ASF did not generate the same positive motor behavior outcomes, it elicited greater subjective motivation and trends toward higher cortical engagement. Together, these results underscore the multidimensional impact of ASF on performance, physiology, and perception, and support the development of adaptive, state-aware VR rehabilitation systems that tailor ASF training to optimize outcomes for individuals with SCI.

## Figures and Tables

**Table 1:** % Change in performance and physiological metrics after training with different conditions of augmented sensory feedback (ASF).

METRIC		% Change in Measure After Training per ASF Condition (1-Sample *t*-Test *p*-Value Versus Zero)		ANOVA Results
Measure	Pre-Training Mean Value	Visual ASF (VF)	Visual Plus Haptic ASF (VHF)	*p*-Value, F-Stat
Performance—pathlength	35.5 m (in VR units)	−12.5 ± 3.3	−1.57 ± 3.25	**7.8 × 10^−4^**, 27.5
		(***p* = 0.0011**)	(*p* = 0.34)	
Performance—comp. time	9.35 s	−12.8 ± 4.5	4.58 ± 15.51	**0.043**, 5.8 *⸙* 0.063
		(*p* = **0.0031**, *⸙* **0.031**)	(*p* = 0.54)	
EEG delta power	155.9 µV^2^/Hz	−52.3 ± 27.8	92.7 ± 205.5	0.16, 2.4 *⸙* 0.063
		(*p* = **0.014**, *⸙* **0.031**)	(*p* = 0.37)	
EEG theta power	10.8 µV^2^/Hz	−9.8 ± 91.1	−9.69 ± 16.0	0.99, 2.4 × 10^−6^ *⸙* 0.63
		(*p* = 0.82)	(*p* = 0.25)	
EEG alpha power	35.2 µV^2^/Hz	−50.7 ± 40.5	−1.41 ± 23.4	**0.046**, 5.6
		(***p* = 0.049**)	(*p* = 0.90)	
EEG beta power	28.4 µV^2^/Hz	−47.3 ± 35.9	14.7 ± 39.8	**0.032**, 6.7
		(***p* = 0.042**)	(*p* = 0.46)	
EMG RMS amplitude	5.4 × 10^−5^ mV	−32.5 ± 13.9	16.2 ± 22.2	**0.0031**, 17.3
		(***p* = 0.0063**)	(*p* = 0.18)	
EDA intra-trial Δ	0.26 µS	−22.5 ± 42.8	126.7 ± 130.2	**0.041**, 5.9
		(*p* = 0.30)	(*p* = 0.095)	
Heart Rate	72.3 beats/min	7.8 ± 10.4	−3.9 ± 3.7	**0.045**, 5.7
		(*p* = 0.17)	(*p* = 0.078)	

Note#1: Reductions (negative shift) in performance metrics indicate improvement. Note#2: ANOVA is one-way for factors across two training conditions—VF, VHF. Note#3: Significant *p*-values < 0.05 marked as bold. Note#4: ‘*⸙*’ indicates results from non-parametric test (Wilcoxon signed ranks).

**Table 2:** Perception metrics regarding training with different conditions of augmented sensory feedback (ASF).

METRIC		% Difference from Participant Mean (1-Sample t-Test *p*-Value Versus Zero)		ANOVA Results
Survey Measure	Mean (0–100)	Visual ASF (VF)	Visual plus Haptic ASF (VHF)	*p*-Value, F-Stat
Agency	82.5 (VF = 82, VHF = 83)	−4.2 ± 6.9	4.2 ± 6.9	0.094, 3.62
		(*p* = 0.25)	(*p* = 0.25)	
Motivation	89.9 (VF = 88, VHF = 92)	−5.6 ± 7.5	5.6 ± 7.5	**0.045**, 5.6
		(*p* = 0.17)	(*p* = 0.17)	
Utility	79.8 (VF = 76, VHF = 84)	−11.7 ± 15.4	11.7 ± 15.4	**0.044**, 5.7 *⸙* 0.063
		(*p* = 0.17, *⸙* **0.31**)	(*p* = 0.17, *⸙* **0.31**)	

Note#1: Reductions (negative shift) in performance metrics indicate improvement. Note#2: ANOVA is one-way for factors across two training conditions—VF, VHF. Note#3: Significant *p*-values < 0.05 marked as bold. Note#4: ‘*⸙*’ indicates results from the non-parametric test (Wilcoxon signed ranks).

## Data Availability

The underlying data for this study will be made available upon request to the corresponding author. The authors will additionally seek to post the data to a publicly available repository with DOI (e.g., ResearchGate).

## Supplementary Materials

The following supporting information can be downloaded at: https://www.mdpi.com/article/10.3390/bioengineering12111266/s1.

## Abbreviations

The following abbreviations are used in this manuscript:

VR	Virtual Reality
SCI	Spinal Cord Injury
ASF	Augmented Sensory Feedback
EEG	Electroencephalography
EMG	Electromyography
HR	Heart Rate
EDA	Electrodermal Activity
VF	Visual Feedback
VHF	Visual plus Haptic Feedback
