# Supplementary material for: Exploratory Investigation of Motor and Psychophysiological Outcomes Following VR-Based Motor Training with Augmented Sensory Feedback for a Pilot Cohort with Spinal Cord Injury
Source: Bioengineering (Basel). 2025 Nov 18;12(11):1266. doi: 10.3390/bioengineering12111266 (PMC12650409; doi:10.3390/bioengineering12111266)
Supplement: Supplementary file 1 [file bioengineering-12-01266-s001.zip › bioengineering-3902712-supplementary.pdf]

Minimal Data Set

participant	perf-path_VF	perf-path_VHF	perf-time_VF	perf-time_VHF	eeg-delta_VF	eeg-delta_VHF	eeg-theta_VF	eeg-theta_VHF	eeg-alpha_VF	eeg-alpha_VHF	eeg-beta_VF	eeg-beta_VHF	emg-amp_VF	emg-amp_VHF	eda-range_VF	eda-range_VHF	ecg-bpm_VF	ecg-bpm_VHF
1	0.8844	0.9922	0.9215	1.251	0.9573	2.007	0.9902	1.072	0.797	0.9571	0.7377	1.02	0.5566	1.331	1.227	1.486	1.205	1.014
2	0.8262	0.9303	0.9039	0.9669	0.4734	0.6643	0.3799	0.6639	0.5709	0.6198	0.4819	0.6301	0.7076	1.177	0.4902	0.9194	0.9452	0.9693
3	0.8658	1.017	0.8262	0.8567	0.2829	5.464	0.1215	0.8456	0.107196	1.104	0.1005244	1.152	0.8333	0.7776	0.4505	1.79	1.002	0.9679
4	0.9183	0.9981	0.8251	1.15	0.311	0.8387	0.5914	0.9172	0.2524	1.251	0.4676	1.196	0.5068	1.252	1.259	4.178	1.127	0.9137
5	0.8803	0.9836	0.8853	1.006	0.3622	0.6613	2.429	1.017	0.917	0.9975	0.9548	1.736	0.7682	1.273	0.4483	2.96	1.111	0.9389
